# Prognostic Implications of DNA Repair, Ploidy and Telomerase in the Malignant Transformation Risk Assessment of Leukoplakia

**DOI:** 10.31557/APJCP.2020.21.2.309

**Published:** 2020

**Authors:** Gigi Thomas, Santhoshkumar TR, Preethi George S, Thara Somanathan, Santhi Sarojam, Nandakumar Krishnankutti, Hariharan Sreedharan, Ravindran Ankathil

**Affiliations:** 1 *Division of Community Oncology, *; 2 *Division of Cancer Epidemiology and Biostatistics, *; 4 *Division of Cytopathology, *; 7 *Division of Cancer Research, Regional Cancer Centre, Medical College Campus, *; 2 *Division of Cancer Research, Rajiv Gandhi Centre for Biotechnology, *; 5 *Research Associate, Child Development Centre, Medical College Campus, Thiruvananthapuram, *; 6 *Dean, Azeezia Dental College, Meyannoor, Kollam, Kerala, India, *; 8 *Human Genome Centre, School of Medical Sciences, Health Campus, University Sains Malaysia, 16150, KubangKerian, Kelantan, Malaysia. *

**Keywords:** Oral Squamous Cell Carcinoma, aneuploidy, diploid, DNA repair capacity, telomerase, Leukoplakia

## Abstract

**Background:**

Although leukoplakia shows a higher risk for malignant transformation to oral cancer, currently there are no clinically relevant biomarker which can predict the potentially high risk leukoplakia. This study aimed to investigate the genetic alterations such as DNA ploidy, telomerase expression and DNA repair capacity as predictive markers of malignant transformation risk of leukoplakia.

**Methods:**

The study was initiated in September 2005 and patients were followed up to March 2014. Two hundred patients with oral leukoplakia, 100 patients with oral cancer and 100 healthy, age and sex matched adults with normal oral mucosa as controls were recruited. The DNA ploidy content was measured by high resolution flow cytometry, level of telomerase expression was identified by TRAP assay and intrinsic DNA repair capacity was measured by mutagen induced chromosome sensitivity assay of cultured peripheral blood lymphocytes. The Chi-square test or Fisher’s Exact test was used for comparison of categorical variables between biomarkers. A p value less than or equal to 0.05 was considered as statistically significant. Analysis was performed with SPSS software version 16. Logistic regression was used to find the association between the dependent and three independent variables.

**Results:**

There was significant difference in the distribution of ploidy status, telomerase activity and DNA repair capacity among control, leukoplakia and oral cancer group (p<0.001). When the molecular markers were compared with histological grading of leukoplakia, both DNA ploidy analysis and telomerase activity showed statistical significance (p<0.001). Both aneuploidy and telomerase positivity was found to coincide with high-risk sites of leukoplakia and were statistically significant (p<0.002 and 0.02). All the leukoplakia and oral cancer patients were followed up for 8.5 years. Out of 200 cases of leukoplakia, 19 were found to be malignancy at the time of diagnosis. So for the calculation of risk of malignant transformation only 181 cases of leukoplakia were considered. Out of this 181 cases of leukoplakia, 12 (6.6%) underwent malignant transformation over a period ranging from 6 to 36 months. Univariate analysis showed DNA repair capacity and DNA ploidy as markers having significant risk association whereas multivariate analysis showed DNA repair capacity as the most significant marker to assess malignant transformation risk of leukoplakia (OR 52.9, 95% CI, 6.56 – 426.7).

**Conclusion:**

Genomic markers combined with clinical and histopathological evaluation can help identify the potentially high risk leukoplakia which can then be treated based on an individual approach.

## Introduction

Leukoplakia is defined as a white plaque of questionable risk having excluded other known disorders that carry no increased risk of cancer (Warnakulasuriya et al., 2007). Leukoplakia may be clinically categorised as homogenous or non-homogenous types. All clinically diagnosed leukoplakia warrant biopsy to exclude underlying carcinoma and dysplasia.

Histopathologically, lesions with higher grades of dysplasia have higher risk of malignant transformation compared to lesions with no dysplasia. It has been observed that some of the dysplastic lesions remain clinically unchanged, while malignant transformation may occasionally take place in non-dysplastic lesions. Hence newer methods for predicting the malignant potential of leukoplakia has to be developed. Since malignant transformation is due to genetic damage over time, identification of genetic changes in Oral Potentially Malignant Disorders (OPMD) such as leukoplakia will be useful for predicting lesions at a risk for malignant transformation. 

However, there is currently no single or set of clinically relevant and useful biomarkers that can readily predict OPMD that could progress to invasive cancer in the absence of intervention (Smith et al., 2009). A panel of markers would increase the probability of identifying malignant transformation potential of high risk leukoplakia. This study was designed to investigate the utility of three genomic alterations as candidate markers that may fulfill these requirements. 

Transformation from a normal cell to a malignant cell may be associated with changes in total DNA content or ploidy. A normal cell has a diploid DNA content (chromosome number of 2N). A cell with numerical chromosome abnormalities such as losses or gains of chromosomes is designated as an aneuploid cell. Numerical aberrations in chromosomes, referred to as aneuploidy and tetraploidy can be observed in human cancers and pre-cancers (Sen, 2000). OPMD which contain DNA aneuploid / tetraploid clones are suspicious of bearing an inherent malignant potential. DNA flow cytometry is a cell based approach that allows rapid and reliable identification of DNA ploidy in cells. 

The occurrence of oral precancerous lesions is related with not only carcinogenic environmental factors, but also some self-regulatory mechanisms. Individual susceptibility to carcinogens, an important determinant of disease risk, is influenced by host factors such as the ability to repair DNA lesions. Genetic factors determine an individual’s DNA repair capability. DNA repair is a system of defenses designed to protect the integrity of the genome. Reduced DNA repair capacity is a significant risk-factor for cancer and can be measured by mutagen-induced chromosome sensitivity assay.

Malignant transformation of normal mucosa is associated with genetic changes that affect cell cycle, apoptosis, angiogenesis and telomere length. Telomerase expression results in direct tumorigenic conversion of normal human epithelial cells and fibroblasts. But the exact point in which the telomerase activation occurs, whether it is expressed at the precancerous or in-situ stage or late in the process of carcinogenesis, is not known. So the ability to detect telomerase activity in the oral precancerous lesions may allow detection of oral cancer at an early stage.

Majority of studies using molecular markers have been conducted in the Western countries where the incidence of oral precancers and the risk of malignant transformation is much lesser compared to the Indian population. As not much work on this aspect has been carried out in India, the present study was undertaken to investigate the genetic alterations such as DNA ploidy content, telomerase expression level and DNA repair capacity in oral leukoplakia and to correlate the findings with histopathological and clinical outcome for a follow period of 8.5 years. For comparison of data, these three candidate markers were investigated in oral cancer patients and normal healthy individuals. 

## Materials and Methods

This study was initiated in September 2005 and the patients were followed up to March 2014. Patients who came to the Community Oncology division of Regional Cancer Centre, Thiruvananthapuram, India, with diagnosis of leukoplakia and oral squamous cell carcinoma were included. Two hundred patients with histopathological diagnosis of leukoplakia, 100 patients with oral squamous cell carcinoma, 100 age and sex matched healthy adults with fibroma, fibroepithelial polyp, pyogenenic granuloma who were willing for excision were included as controls. Informed consents were obtained from subjects. 

 Three sections of tissues were removed from the leukoplakia and oral cancer patients and controls. One section of tissue was used for histopathological examination. Grading of dysplasia was done according to W H O (2005) as mild, moderate and severe dysplasia (Branes et al., 2006). The second and third sections were used for ploidy analysis and telomerase activity. Patients with leukoplakia were on regular follow up. For studying the DNA repair capacity, ten ml blood was collected. 

*Quantification of DNA ploidy by flow cytometry*

**Figure 1:**
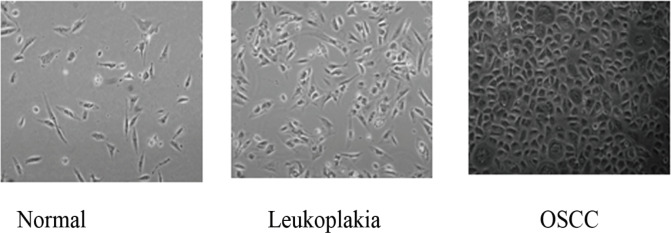
Characterization of Cell Culture: The biopsy samples were washed with HBSS and trypsinised and allowed to grow in culture dishes for one- three weeks as described in materials and methods. The cells expanded from primary cultures were imaged using phase contrast microscope. Representative images showing normal, leukoplakia and cancer cells growing in culture are shown

Primary cultures were established from the biopsy samples for performing ploidy analysis by FACS. A major advantage of this approach is that few fibroblasts cells expanded in culture could be used as an internal control for normal G0/G1 peak determination in flow cytometer histogram. For cell isolation, biopsy samples collected in sterile Hanks Balanced Salt solution (HBSS) washed, cut in small pieces and digested with 0.1% collagenase and 0.1 % trypsin EDTA for 2- 4 hours with occasional agitation at 37° C. The single cell suspension obtained was washed with 10% Fetal Bovine Serum containing media and re-suspended in MEBM containing 20% Fetal Bovine Serum. Cell suspension was seeded on 6 well plates and allowed to grow for two to three weeks (Figure 1). 

*Ploidy Analysis by Hoechst staining*

Cell monolayer washed with PBS EDTA, trypsinized to get single cell suspension and stained with 1µg/ml of Hoechst 33323 dye for 15 minutes in serum free RPMI medium. Cell suspension filtered through 30 µm cell strainer was analyzed using FACS Aria1 equipped with 355nm UV laser . Doublet cells were eliminated from analysis by gating the single cell population utilizing Hoechst Width signal against Hoechst Area. Hoechst intensity histogram of area signal was further analysed for ploidy status.

*Ploidy analysis by Propidium Iodide staining*

**Figure 2:**
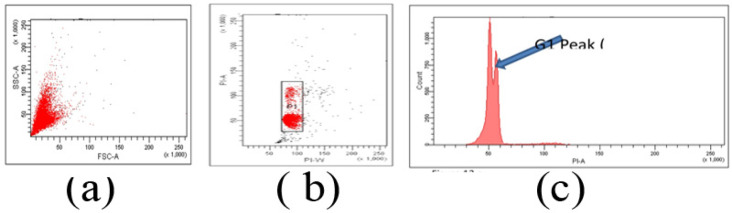
Cell Cycle Analysis by Flow Cytometry. (a), forward scatter signal against side scatter signal; (b), Gate P1 represent singlet population; (c), All cell cycle analysis carried out using the P1 gated cells in PI area histogram. Very close to the G1 peak, an additional peak indicating aneuploidy

Single cell suspension fixed in 70% ethanol for 45 min at 4°C followed by RNase treatment for 30 minutes at 37°C. 10 µg /ml Propidium Iodide added to cell suspension, incubated for 10 minutes in dark at 37°C. Cell suspension filtered through 30 µm cell strainer, analyzed using FACS Aria1SORP System using 488nm laser line. Singlet cells selected based on PI area against PI width signal (Figure 2 a and b). The first population, P1 with lower width represented the singlet population (Figure 2b). This popualtion gated was further analysed in histogram of PI area and was analysed using FACS DIVA software (Figure 2c).

*Telomeric Repeat Amplification Protocol (TRAP) Assay*

PCR-based telomerase activity detection method, TRAP assay was used to detect telomerase activity. For this, tissue samples were washed with PBS and then minced and extracted in ice-cold CHAPS lysis buffer consisting of 10 mMTris-HCl ,1 mM MgCl_2_, 1 mM EGTA, 0.1 mM benzamidine, 5 mM β-mercaptoethanol, 0.5% CHAPS, and 10% glycerol, 200 u/ml of RNase inhibitor by homogenization. Protein concentration was estimated by Bradford method. Telomerase activity in the samples were assayed using TRAPEZE Gel-Based Telomerase Detection Kit as described by Kim et al., (1994) using TRAPEZE Telomerase Detection Kit. Briefly 1μg of protein was mixed with TRAP PCR mixture for PCR amplification. PCR products were electrophoresed on a 12.5% non-denaturing polyacrylamide gel and stained with Silver Nitrate for direct visualization.

*Mutagen induced chromosome sensitivity assay for evaluation of DNA repair capacity*

Employing mutagen induced chromosome sensitivity assay of Hsu et al., (1991), peripheral blood lymphocytes of study subjects were cultured to assess DNA repair capacity with bleomycin as mutagen. Two parallel cultures (A and B ) were set up for each sample. Total incubation time was 72 hours, 37^o^C. Bleomycin treatment (0.03 units/ml) was given during the last 5 hours in culture B ensuring that damage induced in late S and G2 phases of cell cycle could be evaluated at metaphase. During the last one hour, cultures treated with colcemid (0.04 mg/ml) to accumulate mitoses before being harvested for conventional air dried preparations. All Cultures were harvested by standard cytogenetic procedures (Moorhead et al., 1960).Chromosome preparations were stained with Geimsa. 

Chromosome breaks were scored on 100 metaphases per sample. Only frank chromatid breaks or exchanges recorded. Each chromatid break was recorded as one break and each chromatid exchange was recorded as 2 breaks. (Figure 4).The mean number of break per cell (b/c), based on evaluation of 100 metaphases taken as measure of mutagen sensitivity. Any individual expression greater than or equal to 0.8 as mean b/c value was considered sensitive to Bleomycin induced chromosome damage and any individual expression above 1.0 as the mean b/c value was considered mutagen hypersensitive (Spitz et al., 1989). The higher the number of chromosome breaks, poorer is the DNA repair competence. For evaluating DNA repair capability, leukoplakia, oral cancer and controls were grouped into hyposensitive, sensitive and hypersensitive based on statistical significance of mean Bleomycin induced chromosome breaks (b/c) values.

*Data Management and Statistical Analysis*

To find the association between the dependent and independent variables, Pearson Chi-square test was used. Logistic regression was used to find the association between the dependent and three independent variables. Malignant transformation risk of the three candidate genomic markers was analyzed for risk estimation by logistic regression procedure. Sensitivity and specificity analysis of independent markers and combination of markers were also done. A p value ≤ 0.05 was considered as statistically significant. 

## Results

Study was initiated in Regional Cancer Centre, Trivandrum in September 2005 and patients followed up to March 2014. Out of 400 participants, 254 were males (63.5%) and 146 females (36.5%). Mean age of patients was 54 years (SD 12.7). Three groups included were leukoplakia (n =200), oral cancer (n=100) and the control (n =100). 

Significant difference in distribution of ploidy status, telomerase activity and DNA repair capacity among control, leukoplakia and oral cancer group was observed (p<0.001) (Table 1). 

When histologic grading of leukoplakia was compared with DNA ploidy (marker 1) lesions with greater clinical severity had higher grades of dysplasia and abnormal DNA content (Table 2). 

Histologic severity of leukoplakia was associated with telomerase positivity (Marker 2). Greater proportion of telomerase negative cases was found in homogenous leukoplakia (Table 3).

**Figure 3:**
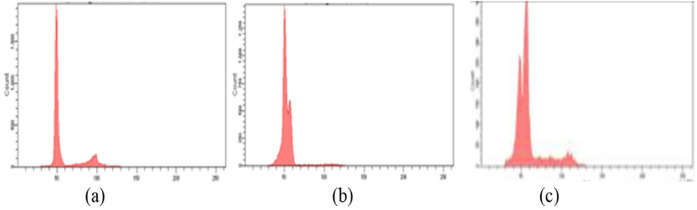
Representative Histogram Showing. a, Diploidy in normal controls; b, Aneuploidy in leukoplakia; c, Aneuploidy in oral squamous cell carcinoma

When the molecular markers were compared with histological grading of leukoplakia, both DNA ploidy and telomerase showed statistical significance (p<0.001). When marker 1 was used, 76.7 % of mild dysplasia followed by 60.7 % moderate dysplasia showed diploidy while aneuploidy was reported in 75% of severe dysplasia and 39.3% in moderate dysplasia (Figure 3). When marker 2 was compared with histologic grading, 93.1% of moderate dysplasia and 87.5% of severe dysplasia showed telomerase positivity. However, no statistically significant difference was observed when marker 3 (DNA repair capacity) was compared with histopathologic diagnosis (p=0.18)

Both aneuploidy and telomerase positivity was found to coincide with high-risk sites of leukoplakia and were statistically significant (p<0.002 and 0.02). All the leukoplakia and oral cancer patients were followed up for 8.5 years. 

Out of 200 cases of leukoplakia, 19 were malignant at time of diagnosis. So for the calculation of risk of malignant transformation only 181 cases of leukoplakia were considered. Out of 181 cases of leukoplakia, 12 (6.6%) underwent malignant transformation over a period ranging from 6 to 36 months (Figures 5a and b). Among these 12 patients ,11 (91.6%) showed increased chromosome hypersensitivity to Bleomycin while ten (83%) patients were telomerase positive and 8/12 (66.7%) showed aneuploidy. Out of the remaining 169 cases 91(54%) of the lesions regressed while in 78 (46%), the lesions remained the same size.

In the univariate analysis using logistic regression, DNA repair capacity and DNA ploidy emerged as markers with significant values to assess the malignant transformation of leukoplakia (Table 4). However, in multivariate analysis using logistic regression analysis only DNA repair capacity emerged as the most significant marker to assess malignant transformation risk of leukoplakia (OR 52.9, 95% CI, 6.56 – 426.7).The sensitivity and specificity analysis of independent markers and a combination of markers also analyzed. DNA repair capacity had the maximum sensitivity (91.67%) and specificity (84.02%) among all three markers (Table 5). 

**Table 1:** Distribution of the Three Candidate Genomic Markers in Leukoplakia and Oral Cancer Compared to Controls

Candidate genomic markers	Control (%)	Leukoplakia (%)	Oral cancer (%)	p-value
Ploidy status				
Diploid	84 (89.4)	125 (67.6)	18 (33.7)	<0.001*
Aneuploid	10 (10.6)	60 (32.4)	68 (79.1)	
Telomerase activity				
Negative	100 (100)	81 (40.5)	0	<0.001*
Positive	0	119 (59.5)	100 (100)	
DNA repair capacity				
Hypo	100 (100)	152 (76)	28 (28)	<0.001*
Sensitive	0	4 (2)	6 (6)	
Hyper	0	44 (22)	66 (66)	

*p-value statistically significant

**Table 2:** Comparison of Ploidy Analysis with Histological Grade

Clinical diagnosis	Ploidy analysis	Hyperkeratosis, Hyperplasia, Parakeratosis, Acanthosis (%)	Inflammatory (%)	Mild dysplasia (%)	Moderate dysplasia (%)	Severe dysplasia (%)	Atypia (%)	Carcinoma (%)
Homogenous leukoplakia (n =91)	Diploid	22 (24.1)	0 (0)	23 (25.2)	6 (6.5)	0 (0)	7 (7.6)	0 (0)
	Aneuploid	2 (2.1)	0 (0)	9 (9.8)	3 (3.2)	2 (2.1)	4 (4.2)	3 (3.2)
Ulcerated leukoplakia (n=68)	Diploid	15 (22)	2 (2.9)	20 (29.4)	8 (11.7)	0 (0)	3 (4.4)	0 (0)
	Aneuploid	0 (0)	0 (0)	3 (4.4)	2 (2.9)	3 (4.4)	4 (5.8)	5 (7.3)
Nodular leukoplakia (n = 18)	Diploid	1 (5.5)	0 (0)	1 (5.5)	3 (16.6)	2 (11.1)	0 (0)	1 (5.50
	Aneuploid	0 (0)	0 (0)	0 (0)	4 (22.2)	0 (0)	0 (0)	5 (27.7)
Verrucous leukoplakia (n =25)	Diploid	8 (32)	0 (0)	2 (8)	1 (4)	0 (0)	2 (8)	0 (0)
	Aneuploid	0 (0)	0 (0)	2(8)	2 (8)	1 (1)	4 (16)	2 (8)

**Table 3:** Comparison of Histologic Grading of Leukoplakia with Telomerase Expression

Clinical diagnosis	Telomerase activity	Hyperkeratosis, Hyperplasia, Parakeratosis, Acanthosis (%)	Inflammatory (%)	Mild dysplasia (%)	Moderate dysplasia (%)	Severe dysplasia (%)	Atypia (%)	Carcinoma (%)
Homogenous leuokoplakia n =91	Negative	25 (27.4)	0	20 (21.9)	3 (3.2)	0	3 (3.2)	1 (1)
	Positive	2 (2.1)	0	14 (15.3)	6 (6.5)	2 (2.1)	10 (11)	5 (5.4)
Ulcerated Leuokoplakia n=68	Negative	11 (16.1)	2 (2.9)	9 (13.2)	0	0	0	0
	Positive	4 (5.8)	0	16 (23.5)	11 (16.1)	3 (4.4)	7 (10.2)	5 (7.3)
Nodular Leuokoplakia n=18	Negative	1 (5.5)	0	0	0	1 (5.5)	0	0
	Positive	1 (5.5)	0	1 (5.5)	7 (38.8)	1 (5.5)	0	6 (33.3)
Verrucous Leuokoplakia n=25	Negative	4 (16)	0	2 (8)	0	0	1 (4)	0
	Positive	4 (16)	0	3 (12)	3 (12)	1 (4)	5 (20)	2 (8)

**Figure 4:**
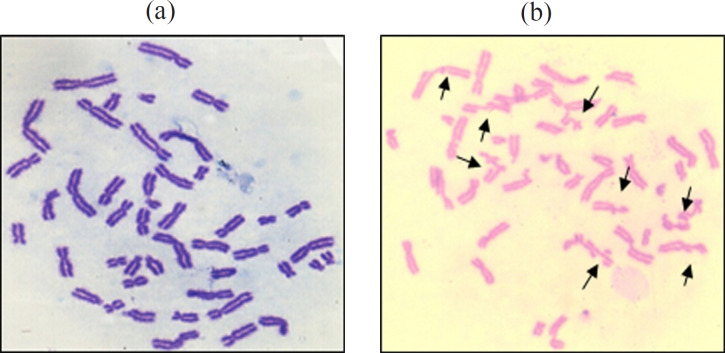
(a), Normal metaphase; (b), Metaphase showing multiple breaks

**Table 4:** Univariate Regression Analysis of Malignant Transformation of Leukoplakia

Candidate Genomic Markers	Malignant Transformation Positive		Malignant Transformation Negative		OR (95% CI) P value	
	No	%	No	%		
Telomerase						
Positive	10	83.33	91	53.8	4.3 (0.91-20.15)	0.0654
Negative	2	16.7	78	46.2		
DNA Repair						
Hypo	1	8.3	142	84.02	57.9 (7.2-466.8)	0.0001*
Hyper	11	91.7	27	15.9		
Ploidy status						
Diploid	4	33.3	120	76.4	6.5 (1.9-22.8)	0.0035*
Aneuploid	8	66.7	37	23.6		

*p-value statistically significant

**Figure 5:**
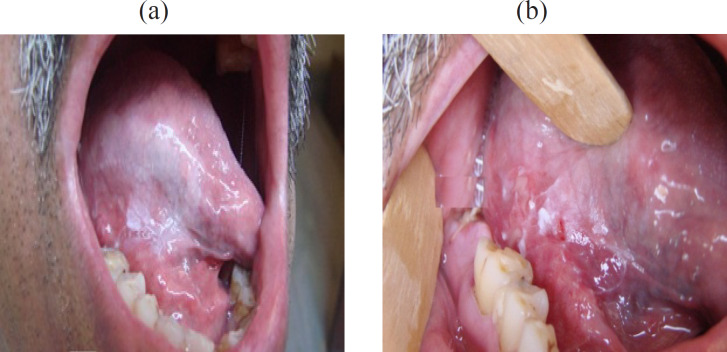
a, Homogenous Leukoplakia of the right lateral margin tongue; b, Malignant transformation on the 18^th ^month of follow-up

**Table 5:** Sensitivity and Specificity of Single and Combination of Genomic Markers

Genomic Markers	Sensitivity (95% CI)	Specificity (95% CI)	Positive Likelihood Ratio (95% CI)	Negative Likelihood Ratio (95% CI)
DNA repair capacity	91.67% (61.52 -99.79)	84.02% (77.61-89.20)	5.74 (3.90-8.44)	0.1 (0.02-0.65)
Telomerase Activity	83.33% (51.59-97.91)	46.15% (38.47-53.98)	1.55 (1.16-2.07)	0.36 (0.10-1.29)
DNA Ploidy	66.67% (34.89-90.08)	77.30% (70.10-83.49)	2.94 (1.80-4.80)	0.43 (0.19-0.96)
DNA repair capacity and DNA Ploidy	66.67% (34.89-90.08)	92.90% (87.93-96.28)	9.39 (4.77-18.47)	0.36 (0.16-0.80)
DNA repair capacity and Telomerase	75.00% (42.81-94.51)	85.80% (79.61-90.68)	5.28 (3.22-8.66)	0.29 (0.11-0.78)
Telomerase and DNA Ploidy	58.33% (27.67-84.83)	82.84% (76.29-88.20)	3.4 (1.90-6.08)	0.5 (0.26-0.99)
DNA repair, Telomerase and DNA Ploidy	58.33% (27.67-84.83)	93.49% (88.65-96.71)	8.96 (4.25-18.88)	0.45 (0.23-0.87)

## Discussion

In India, due to chronic tobacco usage, about 70% oral cancers arise from OPMD, such as leukoplakia. To the best of available knowledge, this is the first study to investigate the impact of a panel of three genomic markers in predicting malignant transformation risk of leukoplakia with a long follow-up period of 8.5 years.

A new technique of DNA ploidy analysis was improvised on fresh tissue samples. Microscopic observation before ploidy analysis confirmed abundance of epithelial cells and occasional fibroblasts at the time of sample processing for DNA analysis (Figure 1). Existence of fibroblasts served as internal control to determine accurate ploidy status in histogram. This technique of first culturing tissue specimens, allowing cells to grow and performing ploidy analysis has not been previously described in the literature. The advantage of this method is its reliability and high yield of cells. However, 7.5% of samples were discarded as they failed to yield epithelial cell colonies due bacterial and yeast contamination. Hence DNA ploidy analysis was done only on 185 / 200 leukoplakia. 

Highest aneuploidy (79.1%) was seen in oral cancer, followed by leukoplakia (32.4%) and least among controls (10.6%) (Table1). Statistically significant difference in ploidy status observed among the three groups, is in agreement with the studies by Khanna et al., (2010) and Vijayavel and Aswath (2013). In this study, DNA ploidy was found to correlate well with clinical findings and histologic criteria. Greater proportion of aneuploidy, ie, 13.1%, 22.2% and 28% of the high grade dysplastic cases of ulcerated, nodular and verrucous leukoplakia respectively were aneuploid (Table 2). Likewise, 77% of non-dysplastic or mild dysplastic cases were diploid while majority (75%) of high grade dysplastic leukoplakia was aneuploid. Present study findings are consistent with the findings of Van Zyl et al., (2012) and Spernadio et al., (2013). In the study by Spernadio, 85 % of the non-dysplastic or mild dysplastic cases were diploid, while most of the moderate or severe dysplastic cases were aneuploid.

The second genomic marker investigated was telomerase expression. In this study, all the subjects in the control arm were telomerase negative and in the oral cancer were telomerase positive (Table 1). On the contrary, 60% of the oral precancers in this study were telomerase positive and is in agreement with the study by Kannan et al., (1996) and Ries et al., (2001) who reported 75% and 50% telomerase positivity. But it differs from the study by Kannan et al., (1996) who detected telomerase activity in normal tissues also. These differences in the results could be probably because the normal tissue in the study by Kannan et al., (1996) was selected from the adjacent normal tissue of oral cancer patients while in the current study, the normal tissue was taken from healthy, normal controls. Telomerase activity was also found to correlate well with the histologic grade and the clinical type of leukoplakia. Around 27% of homogenous and non-dysplastic leukoplakia were found to be telomerase negative while 31%, 44% and 36% of the moderate, severe dysplasia, and atypia cases of ulcerated, nodular and verrucous leukoplakia respectively were found to be telomerase positive (Table 3).

Telomerase is known to be expressed in more than 80% of cancers and may play a role in the early transformation process. However, leukoplakia is not considered to be a transformed phenotype. Interestingly, this study revealed that almost 60% of the leukoplakia patients had increased telomerase activity compared to controls (Table 1). This suggest that leukoplakia even though not transformed, acquires key early malignant transformation potential at least in more than half of the patient groups. Proliferating cells are likely to get additional molecular lesions and such genetic alterations could contribute for malignant transformation. It has been shown that several diploid cells requires inactivation of tumor suppressor genes or activation of one or two oncogenes such as Ras along with telomerase to convert them to malignancy.

The third genomic marker studied was intrinsic DNA repair capacity of study subjects. Among patients with oral cancer, 66% showed defective DNA repair capacity followed by leukoplakia (22%) while it was normal in controls. To the best of available knowledge, this seems to be first comparative study that addressed DNA repair capacity of oral cancer, leukoplakia and controls.

Clinically, location of lesions at high risk sites such as lateral margin of tongue, floor of mouth and soft palate has a higher grade of dysplasia (Mortazavi et al., 2014). In this study, when the site of lesion was compared with the DNA ploidy analysis, 49% of tongue lesions were aneuploid compared to only 28% in the buccal mucosa and is consistent with study by Islam et al., (2010). In this study, Telomerase positivity (79%) was found to coincide with high risk sites of leukoplakia and the results were statistically significant and in agreement with a previous study (Miyoshi et al., 1999).

Majority (75%) of leukoplakia, that underwent malignant transformation were non-homogenous leukoplakia and the malignant transformation rate of 6.6% in 6-36 months is in agreement with other hospital-based studies (Sankaranarayan and Somanathan, 2002). On histopathological examination 5/12 (42%) had either mild or no dysplasia, 4/12 (33.3%) had moderate or severe dysplasia while 3/12 (25%) had atypia. In the limited series (12 cases) of leukoplakia that underwent malignant transformation in our study, 5/12 (42%) had either mild or moderate dysplasia. This is in agreement with findings of Holmstrup et al., (2006), who demonstrated that non dysplastic lesions can also become malignant. From the results on genomic markers observed in these 12 patients who underwent malignant transformation, those who are positive for all three markers should be categorized into very high risk group who should be followed up closely so that malignancy can be picked at the earliest. All these 12 patients who developed carcinoma had early disease and were treated either by surgery or radiation. In our study, univariate analysis using logistic regression was carried out to find out which of the three candidate genomic markers are more suitable. DNA repair capacity had the highest odds of assessing the malignant transformation risk among leukoplakia (OR 57.9, 95% CI, 7.2 - 466.8) followed by DNA ploidy in which the odds of assessing malignant transformation risk among leukoplakia was 6.5 (95% CI 1.9 - 22.8).

In the multivariate analysis, DNA repair capacity was found to be the most significant marker for assessment of malignant transformation among leukoplakia. Controls showed hyposensitivity to bleomycin and demonstrated fewer chromosome breaks (<0.8 b/c). This indicates that they have efficient DNA repair capability. The leukoplakia and oral cancer patients who were hypersensitive to bleomycin had b/c value above 1. In this study, patients with oral cancer showed highest chromosome hypersensitivity to bleomycin (66%), followed by leukoplakia (22%), while controls were hyposensitive. Increased chromosome hypersensitivity in head and neck cancer patients have been reported previously (Hsu et al., 1991; Ankathil et al.,1996). Those individuals who have an inherent deficient DNA repair capacity may have an increased risk to develop cancer, especially tobacco or alcohol habitués. The defect in DNA repair capacity of hypersensitive individuals may be in any one of the enzymatic steps of DNA repair. Hence there may be an increased frequency of chromatid breaks which leads to the development of precancerous lesion which may later undergo malignant transformation. This cytogenetic assay is extremely sensitive in that one chromatid break, representing at least one unrepaired DNA double strand break can be detected per genome in each cell examined. This high level of sensitivity cannot be attained by other methods. 

It was found that the combination of DNA repair capacity and Telomerase activity had the highest sensitivity (75%) and specificity (85.80%) when compared with histopathology. The positive likelihood ratio was highest for the combination of DNA repair capacity and DNA ploidy (9.39, 95% CI: 4.77 - 18.47) and the likelihood ratio of the test being positive to identify the malignant transformation risk was lowest for telomerase activity. This may be probably because all the controls were telomerase negative while all the oral cancer cases were telomerase positive and about 60% of the leukoplakia patients were telomerase positive. Telomerase was found to have the lowest specificity (46%).

When individual marker was analyzed for prognostic value, DNA repair capacity had the highest positive likelihood ratio of 5.74 (95% CI-3.90-8.44) and the sensitivity for DNA repair capacity was 91.7% while specificity was 84%. This was followed by DNA ploidy in which the sensitivity was 66.7% and it had a relatively high specificity of 77.3%. The positive likelihood ratio for this marker was 2.94 (95% CI 1.80-4.80). In the study conducted by Bremmer (2011), ploidy analysis had a sensitivity of 54% and specificity of 60% only which is lower than the current study.

Studies using genomic markers should be done on a large cohort of OPMD and if found beneficial, they can help in the early identification of leukoplakia which have the tendency for malignant transformation. Hence it can be employed as a screening test for assessing cancer risk of oral precancerous lesions. This study highlights the importance of genomic markers in identifying the malignant transformation risk of OPMD. Genomic markers combined with oral visual examination and histopathologic evaluation may help in more precise identification of OPMD cases that have propensity for malignant transformation. It may help identify a small group of high risk individuals who need aggressive treatment while the low risk individuals can be kept for follow up at long intervals. Hence, patients with pre cancers can be treated based on an individual approach. This may prevent progression of OPMD to malignancies thereby reducing the morbidity and mortality due to oral cancer. 
